# Supplementary material for: Sarcopenia and Myosteatosis Are Associated with Neutrophil to Lymphocyte Ratio but Not Glasgow Prognostic Score in Colorectal Cancer Patients
Source: J Clin Med. 2022 May 9;11(9):2656. doi: 10.3390/jcm11092656 (PMC9104763; doi:10.3390/jcm11092656)
Supplement: Supplementary file 1 [file jcm-11-02656-s001.zip › jcm-1641477-supplementary.pdf]

Supplementary material

Aro et al: Sarcopenia and myosteatosis are associated with neutrophil to lymphocyte ratio but not Glasgow Prognostic score in colorectal cancer patients

Table S1. Relationships between two-tiered sarcopenia and myosteatosis and systemic inflammatory markers.

		**Myost**	**Sarco**	**CRP**	**Alb**	**NLR**	**Hb**	**IL1R1**	**IL4**	**IL6**	**IL7**	**IL8**	**IL9**	**IL12p70**	**IFNg**	**CXCL10**	**CCL2**	**CCL4**	**Eotaxin**	**PDGFbb**
Myost	r	1,000	0,133	0,058	0,028	0,192	-0,082	-0,081	-0,142	-0,013	-0,107	-0,036	**-,231***	-0,074	-0,183	0,097	-0,066	0,129	-0,100	0,005
	P value		0,213	0,590	0,797	0,072	0,442	0,451	0,185	0,904	0,319	0,738	0,030	0,491	0,086	0,366	0,539	0,227	0,352	0,963
	N	89	89	89	89	89	89	89	89	89	89	89	89	89	89	89	89	89	89	89
Sarco	r	0,133	1,000	-0,016	-0,087	0,113	-0,180	-0,091	-0,022	-0,104	-0,084	-0,087	-0,140	0,002	-0,169	0,032	0,030	-0,006	-0,143	-0,037
	P value	0,213		0,882	0,420	0,293	0,092	0,399	0,837	0,333	0,432	0,418	0,190	0,987	0,112	0,766	0,779	0,957	0,180	0,729
	N	89	89	89	89	89	89	89	89	89	89	89	89	89	89	89	89	89	89	89
CRP	r	0,058	-0,016	1,000	**-,273****	**,299****	**-,274****	**,374****	0,099	**,544****	**,318****	**,391****	0,153	**,231***	0,147	**,306****	0,115	**,259***	-0,113	0,084
	P value	0,590	0,882		0,010	0,004	0,009	0,000	0,357	0,000	0,002	0,000	0,154	0,030	0,169	0,004	0,281	0,014	0,291	0,433
	N	89	89	89	89	89	89	89	89	89	89	89	89	89	89	89	89	89	89	89
Alb	r	0,028	-0,087	**-,273****	1,000	-0,122	**,325****	-0,176	-0,126	**-,351****	-0,179	**-,327****	-0,052	-0,136	-0,112	-0,069	-0,172	-0,106	-0,069	-0,007
	P value	0,797	0,420	0,010		0,257	0,002	0,099	0,238	0,001	0,094	0,002	0,627	0,204	0,296	0,520	0,106	0,325	0,520	0,948
	N	89	89	89	89	89	89	89	89	89	89	89	89	89	89	89	89	89	89	89
NLR	r	0,192	0,113	**,299****	-0,122	1,000	-0,200	**,304****	**,231***	**,327****	**,313****	**,214***	0,162	**,243***	0,093	0,125	0,102	**,286****	-0,030	**,211***
	P value	0,072	0,293	0,004	0,257		0,060	0,004	0,029	0,002	0,003	0,044	0,130	0,022	0,387	0,243	0,343	0,007	0,779	0,047
	N	89	89	89	89	89	89	89	89	89	89	89	89	89	89	89	89	89	89	89
Hb	r	-0,082	-0,180	**-,274****	**,325****	-0,200	1,000	**-,214***	-0,110	**-,286****	-0,141	**-,341****	-0,040	-0,114	-0,108	**-,226***	-0,094	-0,197	-0,027	-0,052
	P value	0,442	0,092	0,009	0,002	0,060		0,044	0,306	0,007	0,186	0,001	0,713	0,286	0,314	0,033	0,382	0,064	0,802	0,626
	N	89	89	89	89	89	89	89	89	89	89	89	89	89	89	89	89	89	89	89
IL1R1	r	-0,081	-0,091	**,374****	-0,176	**,304****	**-,214***	1,000	**,685****	**,589****	**,745****	**,582****	**,624****	**,528****	**,682****	**,309****	0,161	**,593****	0,033	**,586****
	P value	0,451	0,399	0,000	0,099	0,004	0,044		0,000	0,000	0,000	0,000	0,000	0,000	0,000	0,003	0,132	0,000	0,762	0,000
	N	89	89	89	89	89	89	89	89	89	89	89	89	89	89	89	89	89	89	89

		Myost	Sarco	CRP	Alb	NLR	Hb	IL1R1	IL4	IL6	IL7	IL8	IL9	IL12p70	IFNg	CXCL10	CCL2	CCL4	Eotaxin	PDGFbb
IL4	r	-0,142	-0,022	0,099	-0,126	**,231***	-0,110	**,685****	1,000	**,564****	**,752****	**,532****	**,656****	**,425****	**,816****	0,183	0,128	**,399****	0,180	**,609****
	P value	0,185	0,837	0,357	0,238	0,029	0,306	0,000		0,000	0,000	0,000	0,000	0,000	0,000	0,086	0,233	0,000	0,091	0,000
	N	89	89	89	89	89	89	89	89	89	89	89	89	89	89	89	89	89	89	89
IL6	r	-0,013	-0,104	**,544****	**-,351****	**,327****	**-,286****	**,589****	**,564****	1,000	**,569****	**,692****	**,489****	**,407****	**,634****	**,354****	**,311****	**,348****	0,151	**,251***
	P value	0,904	0,333	0,000	0,001	0,002	0,007	0,000	0,000		0,000	0,000	0,000	0,000	0,000	0,001	0,003	0,001	0,159	0,018
	N	89	89	89	89	89	89	89	89	89	89	89	89	89	89	89	89	89	89	89
IL7	r	-0,107	-0,084	**,318****	-0,179	**,313****	-0,141	**,745****	**,752****	**,569****	1,000	**,590****	**,564****	**,539****	**,662****	**,234***	**,218***	**,461****	0,011	**,648****
	P value	0,319	0,432	0,002	0,094	0,003	0,186	0,000	0,000	0,000		0,000	0,000	0,000	0,000	0,028	0,040	0,000	0,919	0,000
	N	89	89	89	89	89	89	89	89	89	89	89	89	89	89	89	89	89	89	89
IL8	r	-0,036	-0,087	**,391****	**-,327****	**,214***	**-,341****	**,582****	**,532****	**,692****	**,590****	1,000	**,439****	**,410****	**,507****	**,310****	**,392****	**,412****	0,116	**,291****
	P value	0,738	0,418	0,000	0,002	0,044	0,001	0,000	0,000	0,000	0,000		0,000	0,000	0,000	0,003	0,000	0,000	0,280	0,006
	N	89	89	89	89	89	89	89	89	89	89	89	89	89	89	89	89	89	89	89
IL9	r	**-,231***	-0,140	0,153	-0,052	0,162	-0,040	**,624****	**,656****	**,489****	**,564****	**,439****	1,000	**,550****	**,695****	**,214***	**,210***	**,375****	0,138	**,434****
	P value	0,030	0,190	0,154	0,627	0,130	0,713	0,000	0,000	0,000	0,000	0,000		0,000	0,000	0,044	0,049	0,000	0,196	0,000
	N	89	89	89	89	89	89	89	89	89	89	89	89	89	89	89	89	89	89	89
IL12p70	r	-0,074	0,002	**,231***	-0,136	**,243***	-0,114	**,528****	**,425****	**,407****	**,539****	**,410****	**,550****	1,000	**,460****	**,295****	0,129	**,283****	0,090	**,247***
	P value	0,491	0,987	0,030	0,204	0,022	0,286	0,000	0,000	0,000	0,000	0,000	0,000		0,000	0,005	0,227	0,007	0,401	0,020
	N	89	89	89	89	89	89	89	89	89	89	89	89	89	89	89	89	89	89	89
IFNg	r	-0,183	-0,169	0,147	-0,112	0,093	-0,108	**,682****	**,816****	**,634****	**,662****	**,507****	**,695****	**,460****	1,000	**,263***	0,129	**,413****	**,272***	**,417****
	P value	0,086	0,112	0,169	0,296	0,387	0,314	0,000	0,000	0,000	0,000	0,000	0,000	0,000		0,013	0,227	0,000	0,010	0,000
	N	89	89	89	89	89	89	89	89	89	89	89	89	89	89	89	89	89	89	89
CXCL10	r	0,097	0,032	**,306****	-0,069	0,125	**-,226***	**,309****	0,183	**,354****	**,234***	**,310****	**,214***	**,295****	**,263***	1,000	0,113	**,322****	0,055	0,153
	P value	0,366	0,766	0,004	0,520	0,243	0,033	0,003	0,086	0,001	0,028	0,003	0,044	0,005	0,013		0,294	0,002	0,609	0,154
	N	89	89	89	89	89	89	89	89	89	89	89	89	89	89	89	89	89	89	89
CCL2	r	-0,066	0,030	0,115	-0,172	0,102	-0,094	0,161	0,128	**,311****	**,218***	**,392****	**,210***	0,129	0,129	0,113	1,000	0,088	**,417****	0,194
	P value	0,539	0,779	0,281	0,106	0,343	0,382	0,132	0,233	0,003	0,040	0,000	0,049	0,227	0,227	0,294		0,412	0,000	0,069
	N	89	89	89	89	89	89	89	89	89	89	89	89	89	89	89	89	89	89	89
CCL4	r	0,129	-0,006	**,259***	-0,106	**,286****	-0,197	**,593****	**,399****	**,348****	**,461****	**,412****	**,375****	**,283****	**,413****	**,322****	0,088	1,000	-0,005	**,379****
	P value	0,227	0,957	0,014	0,325	0,007	0,064	0,000	0,000	0,001	0,000	0,000	0,000	0,007	0,000	0,002	0,412		0,963	0,000
	N	89	89	89	89	89	89	89	89	89	89	89	89	89	89	89	89	89	89	89

		Myost	Sarco	CRP	Alb	NLR	Hb	IL1R1	IL4	IL6	IL7	IL8	IL9	IL12p70	IFNg	CXCL10	CCL2	CCL4	Eotaxin	PDGFbb
Eotaxin	r	-0,100	-0,143	-0,113	-0,069	-0,030	-0,027	0,033	0,180	0,151	0,011	0,116	0,138	0,090	**,272**[*]	0,055	**,417**[**]	-0,005	1,000	-0,073
	P value	0,352	0,180	0,291	0,520	0,779	0,802	0,762	0,091	0,159	0,919	0,280	0,196	0,401	0,010	0,609	0,000	0,963		0,498
	N	89	89	89	89	89	89	89	89	89	89	89	89	89	89	89	89	89	89	89
PDGFbb	r	0,005	-0,037	0,084	-0,007	**,211**[*]	-0,052	**,586**[**]	**,609**[**]	**,251**[*]	**,648**[**]	**,291**[**]	**,434**[**]	**,247**[*]	**,417**[**]	0,153	0,194	**,379**[**]	-0,073	1,000
	P value	0,963	0,729	0,433	0,948	0,047	0,626	0,000	0,000	0,018	0,000	0,006	0,000	0,020	0,000	0,154	0,069	0,000	0,498	
	N	89	89	89	89	89	89	89	89	89	89	89	89	89	89	89	89	89	89	89

Abbreviations: Myost: myosteatosis; r: Spearman's correlation coefficient; Sarc: Sarcopenia.

**. Correlation is significant at the 0.01 level

*. Correlation is significant at the 0.05 level

Table S2. Relationships between the combined sarcopenia-myosteatosis variable and survival.

	DFS			CSS			OS		
	HR	95%CI	p value	HR	95%CI	p value	HR	95%CI	p value
Age (<70 vs. ≥70)	0.96	0.50-1.86	0.904	2.5	1.33-4.71	**0.004**	2.70	1.57-4.65	**<0.001**
Tumor invasion (T1-T2 vs T3-T4)	0.72	0.33-1.58	0.413	0.68	0.55-2.49	0.678	1.22	0.68-2.21	0.507
Nodal metastases (N0 vs. N1-N2)	8.85	3.86-20.3	**<0.001**	6.62	2.84-15.4	**<0.001**	2.74	1.60-4.69	**<0.001**
Distant metastases (M0 vs. M1)	2.90	0.32-26.53	0.346	7.93	4.15-15.2	**<0.001**	5.41	3.09-9.47	**<0.001**
Sarcopenia alone[1]	2.51	1.10-5.75	**0.029**	1.27	0.62-2.58	0.515	1.12	0.60-2.07	0.730
Myosteatosis alone[1]	4.88	1.44-16.5	**0.011**	2.15	0.89-5.18	0.089	2.07	0.96-4.50	0.065
Both sarcopenia and myosteatosis[1]	1.32	0.44-3.86	0.617	1.12	0.49-2.55	0.797	1.49	0.78-2.86	0.230

[1] Compared with patients without sarcopenia or myosteatosis

Table S3. Distribution of clinocopathological factors in myosteatosis and sarcopenia patient groups in stage I-III colorectal cancer.

Factors	Neither sarcopenia nor myosteatosis (n=79)	Sarcopenia only, no myosteatosis (n=76)	Myosteatosis only, no sarcopenia (n=20)	Both sarcopenia and myosteatosis (n=47)	p-value
**Age, mean, years, (± SD)**	66 (±10.8)	72 (±9.6)	73 (±10.0)	77 (±9.3)	**<0.001**
**Age**					
≤70 years (n=76)	43 (62.3%)	21 (31.8%)	4 (26.7%)	8 (20.0%)	**<0.001**
>70 years (n=114)	26 (37.7%)	45 (68.2%)	11 (73.3%)	32 (80.0%)	
**Gender**					
Male (n=101)	42 (60.9%)	36 (54.5%)	6 (40.0%)	17 (42.5%)	0.212
Female (n=89)	27 (39.1%)	30 (45.5%)	9 (60.0%)	23 (57.3%)	
**BMI, kg/m^2**					
<18.5 (n=2)	1 (1.4%)	1 (1.5%)	0 (0.0%)	0 (0.0%)	**<0.001**
18.5-24.9 (n=58)	22 (31.9%)	12 (18.2%)	3 (20.0%)	21 (52.5%)	
25-29.9 (n=79)	19 (27.5%)	43 (65.2%)	6 (40.0%)	11 (27.5%)	
>30 (n=51)	27 (39.1%)	10 (15.2%)	6 (40.0%)	8 (20.0%)	
**Tumor location**					
Proximal colon (n=78)	25 (36.2%)	21 (31.8%)	10 (66.7%)	22 (55.0%)	**0.021**
Distal colon (n=50)	15 (21.7%)	23 (34.8%)	4 (26.7%)	8 (20.0%)	
rectum (n=62)	29 (42.0%)	22 (33.3%)	1 (6.7%)	10 (25.0%)	
**WHO grade**					
Grade 1 (n=55)	20 (29.0%)	16 (24.2%)	5 (33.3%)	14 (35.0%)	0. 235
Grade 2 (n=115)	45 (65.2%)	41 (62.1%)	10 (66.7%)	19 (47.5%)	
Grade 3 (n=20)	4 (5.8%)	9 (13.6%)	0 (0.0%)	7 (17.5%)	
**TNM stage**					
1 (n=42)	18 (26.1%)	8 (12.1%)	5 (33.3%)	11 (27.5%)	0.283
2 (n=75)	27 (39.1%)	27 (40.9%)	5 (33.3%)	16 (40.0%)	
3 (n=73)	24 (34.8%)	31 (47.0%)	5 (33.3%)	13 (32.5%)	
4 (n=0)					

**Depth of invasion**					
T1 (n=13)	7 (10.1%)	3 (4.5%)	1 (6.7%)	2 (5.0%)	0.383
T2 (n=43)	15 (21.7%)	9 (13.6%)	5 (33.3%)	11 (27.5%)	
T3 (n=152)	45 (62.5%)	52 (78.8%)	9 (60.0%)	24 (60.0%)	
T4 (n=14)	2 (2.9%)	2 (3.0%)	0 (0.0%)	3 (7.5%)	
**Nodal metastases**					
N0 (n=117)	45 (65.2%)	35 (53.0%)	10 (66.7%)	27 (67.5%)	0.052
N1 (n=53)	16 (23.2%)	24 (36.4%)	1 (6.7%)	12 (30.0%)	
N2 (n=20)	8 (11.6%)	7 (10.6%)	4 (26.7%)	1 (2.5%)	
**Serrated morphology**					
yes (n=56)	15 (21.7%)	14 (21.2%)	6 (40.0%)	21 (52.5%)	**0.002**
no (n=134)	54 (78.3%)	52 (78.8%)	9 (60.0%)	19 (47.5%)	
**ASA grade**					
I (n=12)	6 (9.0%)	4 (6.2%)	1 (7.7%)	1 (2.8%)	**<0.001**
II (n=78)	39 (58.2%)	30 (46.2%)	1 (7.7%)	8 (22.2%)	
III (n=74)	16 (23.9%)	27 (41.5%)	9 (69.3%)	22 (61.1%)	
IV (n=17)	6 (9.0%)	4 (6.2%)	2 (15.4%)	5 (13.9%)	
**Diabetes**					
No (n=154)	56 (81.2%)	58 (87.9%)	10 (66.7%)	30 (75.0%)	0.158
Yes (n=36)	13 (18.8%)	8 (12.1%)	5 (33.3%)	10 (25.0%)	
**Coronary Artery disease**					
No (n=149)	58 (84.1%)	54 (81.8%)	8 (53.3%)	29 (72.5%)	0.052
Yes (n=41)	11 (15.9%)	12 (18.2%)	7 (46.7%)	11 (27.5%)	
**Use of blood pressure lowering medication**					
No (n=76)	35 (50.7%)	27 (40.9%)	2 (13.3%)	12 (30.0%)	**0.023**
Yes (n=114)	34 (49.3)	39 (59.1%)	13 (86.7%)	28 (70.0%)	
**Use of cholesterol lowering medication**					
No (n=119)	44 (63.8%)	40 (60.%)	6 (40.0%)	29 (72.5%)	0.165
Yes (n=71)	25 (36.2%)	26 (39.4%)	9 (60.0%)	11 (27.5%)	
**CRP, mg/L**					

≤10 (n=160)	57 (82.6%)	55 (83.3%)	15 (100.0%)	33 (82.5%)	0.370
>10 (n=30)	12 (17.4%)	11 (16.7%)	0 (0.0%)	7 (17.5%)	
**Modified Glasgow Prognostic Score**					
0 (n=161)	57 (82.6%)	56 (84.8%)	15 (100.0%)	33 (82.5%)	0.335
1 (n=28)	12 (17.4%)	10 (15.2%)	0 (0.0%)	6 (15.0%)	
2 (n=1)	0 (0.0%)	0 (0.0%)	0 (0.0%)	1 (2.5%)	
**NLR**					
≤3 (n=143)	60 (88.2%)	45 (69.2%)	13 (86.7%)	25 (62.5%)	**0.006**
>3 (n=45)	8 (11.8%)	20 (30.8%)	2 (13.3%)	15 (37.5%)	
**Albumin, g/L**					
≤35 (n= 5)	0 (0.0%)	1 (1.5%)	1 (6.7%)	3 (7.5%)	**0.037**
>35 (185)	69 (100.0%)	65 (98.5%)	14 (93.3%)	37 (92.5%)	
**BRAF VE1 immunohistochemistry**					
Negative (n=165)	62 (89.9%)	61 (92.4%)	11 (73.3%)	31 (77.5%)	**0.045**
Positive (n=25)	7 (10.1%)	5 (7.6%)	4 (26.7%)	9 (22.5%)	

Abbreviations: BMI: body mass index; ASA: American Society of Anesthesiologists; CRP: C-reactive protein; NLR: Neutrophil-Lymphocyte Ratio

Table S4. Serum cytokine and chemokine levels, blood cell counts and other laboratory parameters in relation to sarcopenia and myosteatosis in stage I-III colorectal cancer.

**Factors**	**Neither sarcopenia nor myosteatosis (N=79)**	**Sarcopenia only, no myosteatosis (N=76)**	**Myosteatosis only, no sarcoipenia (N=20)**	**Both sarcopenia and myosteatosis (N=47)**	**p-value**
**Laboratory parameters, median (IQR)**					
Serum CRP, mg/L	2.00 (0.70-7.51)	1.90 (0.76-6.64)	1.69 (0.60-5.76)	3.24 (1.00-6.26)	0.673
Serum Albumin, g/L	44.00 (41.00-45.75)	43.0 (41.00-45.00)	43.0 (40.00-46.00)	42.0 (38.25-45.00)	0.301
Blood leukocytes[1]	6.70 (5.60-7.98)	6.50 (5.20-7.75)	8.10 (6.40-8.70)	6.85 (5.65-7.80)	0.169
Blood neutrophils[1]	3.80 (2.83-5.00)	3.90 (2.80-4.90)	4.80 (3.90-5.80)	4.05 (3.03-5.28)	0.293
Blood lymphocytes[1]	1.90 (1.50-2.40)	1.70 (1.30-2.05)	2.30 (1.70-2.80)	1.80 (1.20-2.30)	**0.034**
Blood NLR	2.05 (1.46-2.54)	2.21 (1.50-3.28)	2.08 (1.64-2.68)	2.54 (1.63-3.40)	**0.048**
Blood monocytes[1]	0.60 (0.44-0.70)	0.55 (0.40-0.70)	0.70 (0.60-0.80)	0.60 (0.50-0.80)	**0.022**
Haemoglobin, g/L	131.0 (116.0-142.3)	129.5 (111.8-140.0)	122.0 (109.0-138.0)	121.0 (109.0-129.0)	**0.010**
CEA, µg/L	1.45 (0.95-3.43)	1.60 (0.95-4.38)	1.60 (1.00-4.10)	2.20 (1.30-5.00)	0.231
**Cytokines, pg/ml, median (IQR)**					
IL-1R1	75.63 (45.83-98.78)	47.94 (33.74-68.39)	46.59 (29.91-98.62)	40.14 (29.39-93.32)	0.321
IL-4	0.95 (0.75-1.14)	0.82 (0.63-1.06)	0.78 (0.56-0.84)	0.86 (0.59-0.93)	0.315
IL-6	5.82 (3.65-8.44)	4.33 (3.06-6.07)	4.58 (3.13-6.59)	4.31 (2.83-10.38)	0.434
IL-7	5.95 (4.04-8.26)	4.97 (3.44-6.54)	5.01 (4.31-6.55)	4.46 (3.24-6.73)	0.353
CXCL8	12.54 (10.30-16.93)	9.22 (8.41-13.58)	11.50 (9.20-12.59)	10.18 (8.44-18.49)	0.278

IL-9	11.11 (6.51-16.64)	7.35 (4.84-11.39)	6.88 (2.94-13.07)	4.73 (3.35-8.44)	**0.037**
IL-12	31.00 (21.10-44.72)	29.75 (11.44-42.39)	31.72 (11.52-37.69)	26.31 (7.87-34.77)	0.719
IFNg	39.43 (31.09-47.84)	27.26 (19.11-38.24)	25.28 (19.61-36.49)	26.53 (19.74-35.16)	0.027
CXCL10	845 (641-1110)	899 (5623-1132)	1068 (651-1706)	877 (697-1354)	0.753
CCL2	13.98 (10.44-19.35)	15.17 (10.56-24.02)	17.51 (12.22-23.56)	11.65 (4.47-21.57)	0.493
CCL4	66.73 (48.74-83.75)	55.11 (47.13-63.49)	58.56 (42.10-101.15)	67.55 (46.17-88.48)	0.395
CCL11	132 (114-183)	123 (92-171)	154 (84-180)	121 (87-171)	0.597
PDGF-BB	9279 (5803-4823)	7678 (5203-10059)	8935 (5105-12863)	7895 (3056-12498)	0.788

Abbreviations: CRP: C-reactive protein; NLR: Neutrophil-Lymphocyte Ratio; CEA: carcinoembryonic antigen; [1]: x 10^9/L;

Table S5. Multivariable regression model for myosteatosis probability in stage I-III colorectal cancer.

**Factors**	**OR**	**95%CI**	**p-value**
Age (<70 vs. ≥70 years)	1.68	0.68–4.19	0.265
Tumor location (proximal vs. distal colon)	0.491	0.18–1.33	0.162
Tumor location (proximal colon vs. rectum)	0.498	0.20–1.24	0.134
Serrated morphology (yes vs. no)	3.54	1.56–8.00	**0.002**
ASA grade (I-II vs. III-IV)	4.42	1.70–11.47	**0.002**
NLR (<3 vs. >3)	1.13	0.47–2.69	0.784
Albumin level (<35 g/L vs. >35 g/L)	0.22	0.02-2.41	0.215
Blood pressure lowering medication (yes/no)	0.92	0.37-2.33	0.868

Abbreviations: OR: odds ratio; CI: confidence interval; ASA: American Society of Anesthesiologists; BMI: CEA: carcinoembryonic antigen; NLR: Neutrophil-Lymphocyte Ratio.

Table S6. Multivariable regression model for sarcopenia probability in stage I-III colorectal cancer.

**Factors**	**OR**	**95%CI**	**p-value**
Age (<70 vs. ≥70 years)	3.92	1.87–8.23	**<0.001**
Tumor location (proximal vs. distal colon)	2.09	0.87–4.99	0.099
Tumor location (proximal colon vs. rectum)	1.37	0.62–3.02	0.430
Serrated morphology (yes vs. no)	1.33	0.63–2.81	0.456
ASA grade (I-II vs. III-IV)	1.22	0.55–2.69	0.624
NLR (<3 vs. >3)	3.17	1.38–7.28	**0.007**
Albumin level (<35 g/L vs. >35 g/L)	0.63	0.06-6.52	0.701
Blood pressure lowering medication (yes/no)	1.15	0.53-2.47	0.729

Abbreviations: OR: odds ratio; CI: confidence interval; ASA: American Society of Anesthesiologists; BMI: CEA: carcinoembryonic antigen; NLR: Neutrophil-Lymphocyte Ratio.

Table S7. Multivariable Cox regression model for overall survival in stage I-III colorectal cancer.

**Factors**	**HR**	**95%CI**	**p-value**
Age (<70 vs. ≥70)	2.49	1.25–4.96	**0.009**
Tumor invasion (T1-T2 vs. T3-T4)	1.08	0.56–2.09	0.822
Nodal metastases (N0 vs. N1-N2)	3.05	1.68–5.55	**<0.001**
Myosteatosis (No vs. Yes)	1.81	1.00–3.26	**0.048**

HR: hazard ratio; CI: confidence interval. Median follow-up time 60 months; 48 (25.3%) events.

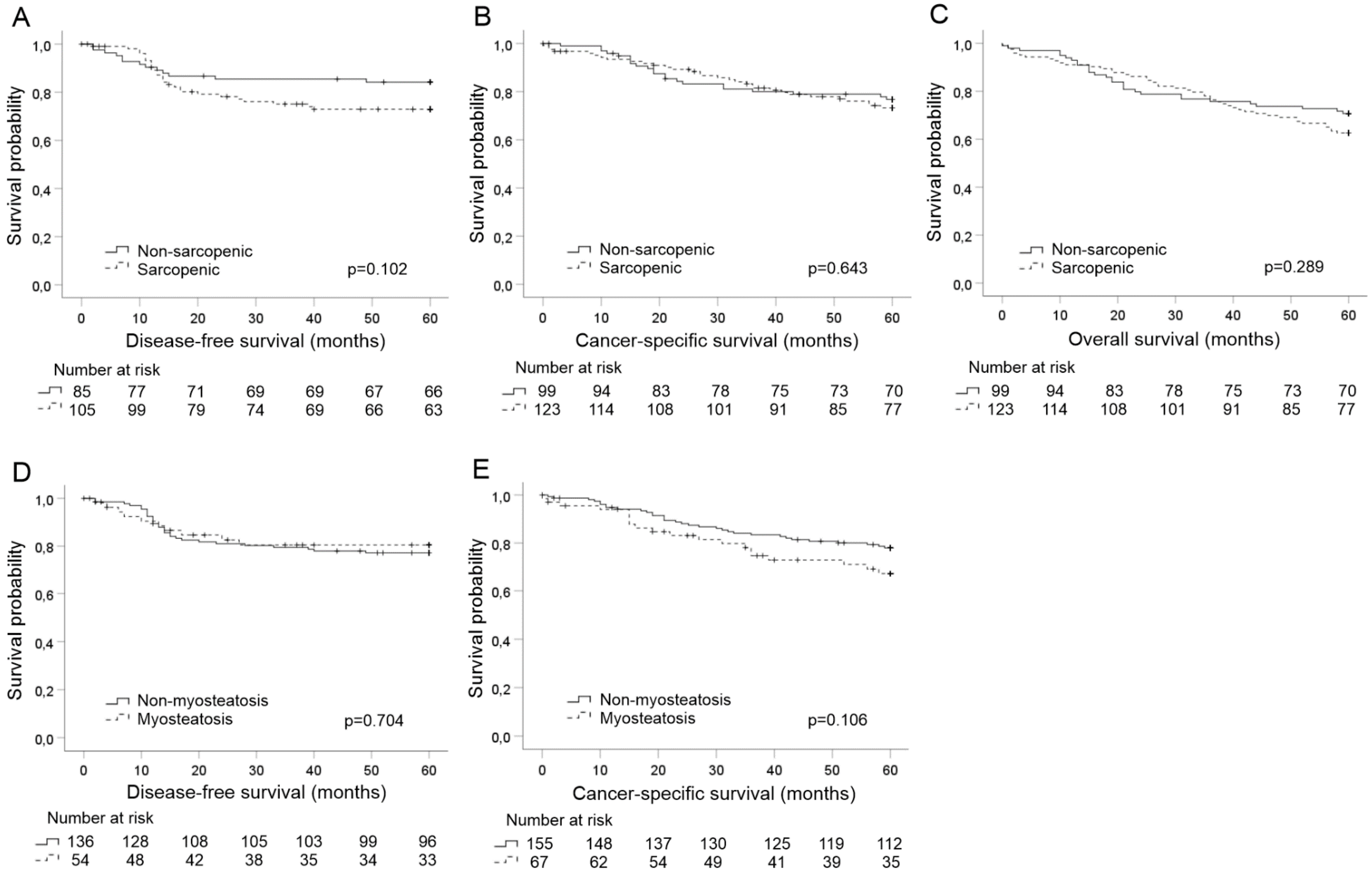

Supplementary Figure S1. Kaplan-Meier survival curves for (**A**) sarcopenia and disease-free survival, (**B**) sarcopenia and cancer-specific survival, (**C**) sarcopenia and overall survival, (**D**) myosteatosis and disease-free survival, and (**E**) myosteatosis and cancer-specific survival.

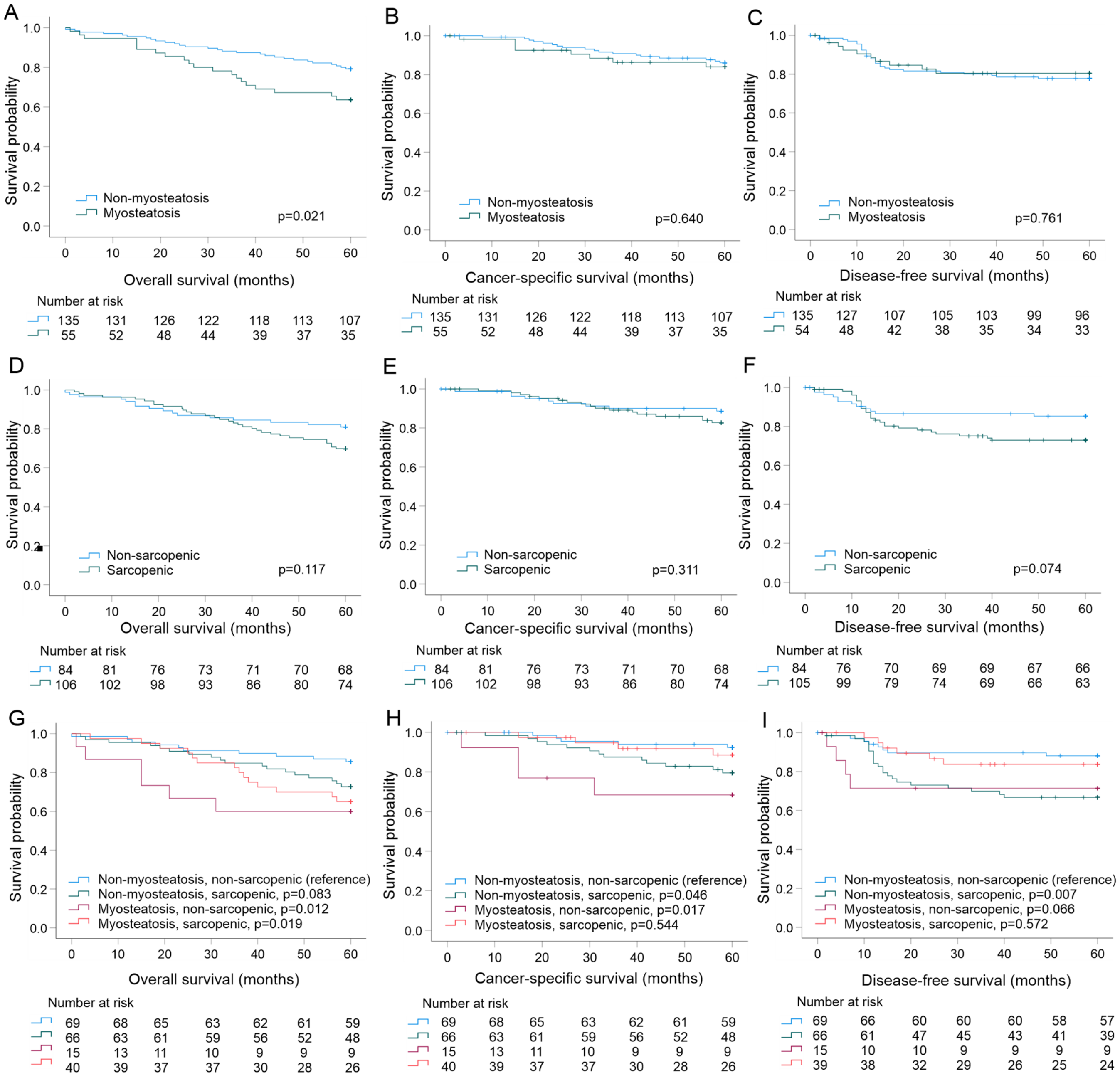

A
Survival probability
Non-myosteatosis
Myosteatosis
p=0.021
Overall survival (months)
Number at risk
135 131 126 122 118 113 107
55 52 48 44 39 37 35
B
Survival probability
Non-myosteatosis
Myosteatosis
p=0.640
Cancer-specific survival (months)
Number at risk
135 131 126 122 118 113 107
55 52 48 44 39 37 35
C
Survival probability
Non-myosteatosis
Myosteatosis
p=0.761
Disease-free survival (months)
Number at risk
135 127 107 105 103 99 96
54 48 42 38 35 34 33
D
Survival probability
Non-sarcopenic
Sarcopenic
p=0.117
Overall survival (months)
Number at risk
84 81 76 73 71 70 68
106 102 98 93 86 80 74
E
Survival probability
Non-sarcopenic
Sarcopenic
p=0.311
Cancer-specific survival (months)
Number at risk
84 81 76 73 71 70 68
106 102 98 93 86 80 74
F
Survival probability
Non-sarcopenic
Sarcopenic
p=0.074
Disease-free survival (months)
Number at risk
84 76 70 69 69 67 66
105 99 79 74 69 66 63
G
Survival probability
Non-myosteatosis, non-sarcopenic (reference)
Non-myosteatosis, sarcopenic, p=0.083
Myosteatosis, non-sarcopenic, p=0.012
Myosteatosis, sarcopenic, p=0.019
Overall survival (months)
Number at risk
69 68 65 63 62 61 59
66 63 61 59 56 52 48
15 13 11 10 9 9 9
40 39 37 37 30 28 26
H
Survival probability
Non-myosteatosis, non-sarcopenic (reference)
Non-myosteatosis, sarcopenic, p=0.046
Myosteatosis, non-sarcopenic, p=0.017
Myosteatosis, sarcopenic, p=0.544
Cancer-specific survival (months)
Number at risk
69 68 65 63 62 61 59
66 63 61 59 56 52 48
15 13 11 10 9 9 9
40 39 37 37 30 28 26
I
Survival probability
Non-myosteatosis, non-sarcopenic (reference)
Non-myosteatosis, sarcopenic, p=0.007
Myosteatosis, non-sarcopenic, p=0.066
Myosteatosis, sarcopenic, p=0.572
Disease-free survival (months)
Number at risk
69 66 60 60 60 58 57
66 61 47 45 43 41 39
15 10 10 9 9 9 9
39 38 32 29 26 25 24

Supplementary Figure S2. Kaplan-Meier survival curves for stage I-III patients (**A**) myosteatosis and overall survival, (**B**) myosteatosis and cancer-specific survival, (**C**) myosteatosis and disease-free survival, (**D**) sarcopenia and overall survival, (**E**) sarcopenia and cancer-specific survival, (**F**) sarcopenia and disease-free survival, (**G**) the presence of myosteatosis and sarcopenia and overall survival, (**H**) the presence of myosteatosis and sarcopenia and cancer-specific survival, (**I**) the presence of myosteatosis and sarcopenia and disease-free survival.
